# Modeling interfacial tension of surfactant–hydrocarbon systems using robust tree-based machine learning algorithms

**DOI:** 10.1038/s41598-023-37933-0

**Published:** 2023-07-05

**Authors:** Ali Rashidi-Khaniabadi, Elham Rashidi-Khaniabadi, Behnam Amiri-Ramsheh, Mohammad-Reza Mohammadi, Abdolhossein Hemmati-Sarapardeh

**Affiliations:** 1 Department of Petroleum Engineering, EOR Research Center, Omidiyeh Branch, Islamic Azad University, Omidiyeh, Iran; 2 Department of Mathematics, Yazd University, Yazd, Iran; 3 Department of Petroleum Engineering, Shahid Bahonar University of Kerman, Kerman, Iran; 4 State Key Laboratory of Petroleum Resources and Prospecting, China University of Petroleum (Beijing), Beijing, China

**Keywords:** Energy science and technology, Engineering, Chemical engineering

## Abstract

Interfacial tension (IFT) between surfactants and hydrocarbon is one of the important parameters in petroleum engineering to have a successful enhanced oil recovery (EOR) operation. Measuring IFT in the laboratory is time-consuming and costly. Since, the accurate estimation of IFT is of paramount significance, modeling with advanced intelligent techniques has been used as a proper alternative in recent years. In this study, the IFT values between surfactants and hydrocarbon were predicted using tree-based machine learning algorithms. Decision tree (DT), extra trees (ET), and gradient boosted regression trees (GBRT) were used to predict this parameter. For this purpose, 390 experimental data collected from previous studies were used to implement intelligent models. Temperature, normal alkane molecular weight, surfactant concentration, hydrophilic–lipophilic balance (HLB), and phase inversion temperature (PIT) were selected as inputs of models and independent variables. Also, the IFT between the surfactant solution and normal alkanes was selected as the output of the models and the dependent variable. Moreover, the implemented models were evaluated using statistical analyses and applied graphical methods. The results showed that DT, ET, and GBRT could predict the data with average absolute relative error values of 4.12%, 3.52%, and 2.71%, respectively. The R-squared of all implementation models is higher than 0.98, and for the best model, GBRT, it is 0.9939. Furthermore, sensitivity analysis using the Pearson approach was utilized to detect correlation coefficients of the input parameters. Based on this technique, the results of sensitivity analysis demonstrated that PIT, surfactant concentration, and HLB had the greatest effect on IFT, respectively. Finally, GBRT was statistically credited by the Leverage approach.

## Introduction

Interfacial tension (IFT) is a parameter of interest in petroleum and chemical science and engineering^1–3^. It plays a vital role in multiphase flow, separation processes, formation and stability of emulsions, fluid transportation, and reservoir engineering processes like fluid contacts, fluid saturation distribution, recovery mechanisms, and enhanced oil recovery (EOR) processes^4–8^.

Capillary pressure plays a critical role in oil recovery at all stages of production from oil reservoirs. The capillary number (*N*_*c*_) concept and equation are used to investigate the effect of capillary pressure on oil recovery from the reservoir. The general form of the capillary number is defined as follows^9, 10^:

1 $${\text{N}}_{\text{c}}{=}\frac{\left(\text{viscous force}\right)}{\left(\text{capillary force}\right)}{=}\frac{\upmu \upnu}{{\upsigma}\,{\text{cos}}\,{\uptheta}}$$

where *θ* is the contact angle, *σ* is the IFT between the wetting and non-wetting phase, *μ* is the viscosity of the displacing phase, and *v* is the Darcy velocity. The amount of oil saturation remaining in the porous medium has a strong correlation with the capillary number^9–15^. Researchers concluded that increasing the capillary number increases oil recovery^16^. Also, capillary number was considered as the primary variable in several modeling and simulation studies related to IFT and wettability alteration^15, 17, 18^. According to Eq. (1), decreasing the IFT increases the capillary number. EOR techniques produce residual oil by optimizing the amount of capillary number^18–21^. Surfactants are amphiphilic molecules that are soluble in both organic solvents and water^22^. The surfactant reduces the IFT between oil and water by adsorbing at the liquid–liquid interface^23, 24^. It was found that an oil droplet on the meniscus could be attracted to the wall when surfactant is added to water, while bubbles always move towards the walls; IFT and gravity play significant roles in these cases^25, 26^. Researchers have conducted numerous laboratory studies to investigate the ability of surfactants to reduce the IFT between aqueous solution and oil for use in EOR techniques^21, 27–29^. The measurement of IFT of a water-hydrocarbon interface in the presence of surfactants is of great interest for surfactant flooding. Various parameters affect the IFT between the solution containing surfactant and hydrocarbons that must be considered. Experimental studies have shown that the type^28, 30^ and the surfactant concentration^31^, the temperature of the aqueous solution^32^, and the hydrocarbon composition^33, 34^ can affect the IFT. Surfactants are divided into two general categories, which include ionic and nonionic surfactants. Ionic surfactants have a positive or negative electric charge, or both, classified into cationic, anionic, and amphoteric surfactants, respectively^35^. However, nonionic surfactants do not have an electric charge. The interfacial behavior of surfactants can vary depending on their structure^2, 35, 36^. Therefore, many researchers have investigated the role of surfactant structure in reducing the IFT between hydrocarbons and aqueous solutions. Strey^37^ showed that with increasing temperature, the IFT behavior of the surfactant is curved and has a minimum point. It was found that before the minimum point of IFT, increasing the temperature which leads to an increase in the number of surfactant molecules at the interface between hydrocarbon and aqueous solution, reduces the IFT value^37^. Also, increasing the surfactant concentration to the critical micelle concentration (CMC) reduces the IFT^35, 38^.

The best way to measure the IFT between surfactant and hydrocarbon is performing laboratory methods. Laboratory methods for measuring IFT are the weight of drop method^39, 40^ pendant drop^41–43^, spinning drop^44–46^, etc. Time-consuming is one of the limitations and challenges of laboratory methods. Considering the price of the chemicals used to perform the IFT test and the cost of the tests, this method is costly. Therefore, developing a model for predicting the IFT between surfactants and hydrocarbons can be very attractive and practical. Previous studies have described the effect of surfactants on the interfacial boundary of two fluids with the surface equation of state^47, 48^. The surface equation of state is a relationship between the surface concentration of surfactant and surface pressure^48^. The difference between the IFT without surfactant and after a surfactant to the solution is equal to the surface pressure^48^. Also, the concentration of surfactant molecules on the surface is defined by surface adsorption^49^. Different approaches to obtaining state equations were discussed in the literature. The Szyszkowski equation^50^, the Frumkin equation^51^, and the Langmuir model^52^ are three examples of semi-empirical equations for the surface equation of state. The Langmuir model was used to describe the adsorption of nonionic surfactants at the interface between hydrocarbons and aqueous solution. This model cannot predict the effect of a surfactant mixture solution on IFT. It is also not suitable for describing the interfacial behavior of surfactants in the presence of inorganic ions^53^. Mulqueen and Blankschtein (2002)^54^ developed a different molecular-thermodynamic approach to predict surfactant adsorption at the oil/water and air/water interfaces. They evaluated the validity of this model only on a limited number of laboratory data, and it was valid only for decane/water interface^54^. Bahramian and Zarbakhsh (2015)^48^ performed studies to estimate the IFT of ionic surfactants. In their proposed model, they considered the size of the surfactant molecule and the CMC value of a surfactant in the aqueous solution as independent variables. In this method, a laboratory test set is required to obtain the CMC^48^. In a recent study, Nikseresht et al. (2019)^55^ used the Butler equation to estimate the IFT between ionic surfactants and normal alkanes as the oil phase. This model is based on the surface state equation. For each case, two fitting parameters need to be set. In other words, the set model is not suitable for other conditions. They also examined Bahramian and Zarbakhsh’s^48^ equation for various surfactants and concluded that it could not be satisfactorily used to predict IFT in the presence of C_10_TAB and C_12_TAB^55^. In summary, thermodynamic models for estimating IFT have the following limitations: (1) they require laboratory testing to calculate the input parameters. (2) Each model fitted to a surfactant does not apply to other cases and conditions. (3) these models were evaluated for limited experimental data. (4) All parameters affecting IFT were not considered in these models. On the other hand, machine learning methods can model and solve complex numerical problems in industry^56–58^. Predicting the IFT between two immiscible fluids using intelligent methods has been considered by many researchers^59–62^. In previous studies, the ability of intelligent methods to estimate the IFT between hydrocarbons and the aqueous solution was evaluated^63–67^. It was found that intelligent methods are appropriate for this purpose. As the literature review shows, the IFT studies in recent years are mostly focused on the aqueous phase and hydrocarbons, and the role of surfactants is less considered. To the best of the authors’ knowledge, no reliable and comprehensive model has been presented for this case. Therefore, there is a window to develop a reliable model for predicting the IFT of surfactants and hydrocarbon systems.

This study aims to develop accurate and reliable models to estimate the IFT between ionic surfactants and normal alkanes. Decision tree (DT), extra trees (ET), and gradient boosted regression trees (GBRT) models are implemented for this purpose. Temperature, normal alkane molecular weight, surfactant concentration, hydrophilic–lipophilic balance (HLB), and phase inversion temperature (PIT) are selected as inputs and independent variables. Also, the IFT between the surfactant solution and normal alkanes is selected as the output and the dependent variable. Sample data are collected from the literature to train and evaluate the models. The trial-and-error method is used to optimize the implemented models. In the present study, the implemented models are evaluated using statistical analysis and applied graphical methods. Furthermore, sensitivity analysis is performed on how changes in the model's inputs impact the IFT values. Finally, the leverage method is carried out to ensure the credibility of the gathered IFT databank and the accuracy and dependability of the best model for estimating the IFT between ionic surfactants and normal alkanes. Hence, the main contributions of this research are as follows:

- Collecting an extensive database of IFT of surfactant–hydrocarbon systems including important parameters such as HLB and PIT_x_, which have a significant impact on the better characterization of surfactants.
- Development of accurate models with low error using robust tree-based machine learning algorithms.
- Performing sensitivity analysis to detect the relative effect of temperature, normal alkane molecular weight, surfactant concentration, HLB, and PIT_x_ on the IFT of surfactant–hydrocarbon systems.
- Implementation of leverage method to identify suspicious and outlier data related to IFT of surfactant–hydrocarbon systems reported in the literature.

## Data gathering

In order to develop the models, 390 sample data were collected from previous studies^68–76^. The sample dataset contains temperature, normal alkane molecular weight, surfactant concentration, HLB, and PIT_x_. In this paper, five different surfactants were used, and their specifications were presented in Table 1. Also, n-hexane, n-heptane, n-octane, n-nonane, n-decane, n-undecane, n-dodecane, n-tetradecane, and n-heptadecane were used as normal alkanes. HLB and PIT_x_ were used to represent the type of surfactants. The HLB value determines the hydrophilicity and lipophilic of a surfactant^77, 78^. Researchers have developed various methods for calculating the amount of the HLB^79, 80^. In this study, the method introduced by Davies (1957)^80^ was used to calculate the HLB. Davis method calculates the value of the HLB based on the group number as follows:

**Table 1:** Characteristics of surfactants used in this study.

Surfactant		Chemical formula	dPIT/dx	HLB
Decyl trimethyl ammonium bromide	C_10_TAB	C_10_H_21_N(CH_3_)_3_Br	338	21
Dodecyl trimethyl ammonium bromide	C_12_TAB	C_12_H_25_N(CH_3_)_3_Br	486	19
Myristyl trimethyl ammonium bromide	C_14_TAB	C_14_H_29_N(CH_3_)_3_Br	453	18
Hexadecyl trimethyl ammonium bromide	C_16_TAB	C_16_H_33_N(CH_3_)_3_Br	426	17
Sodium dodecyl sulfate	SDS	C_12_H_25_NaSO_4_	499	40

2 $$\text{HLB }_{\text{Davies}} = 7 + {\Sigma} \, (\text{hydrophilic group numbers}) - \, { \Sigma} (\text{lipophilic group numbers}{)}$$

**Figure 1:**
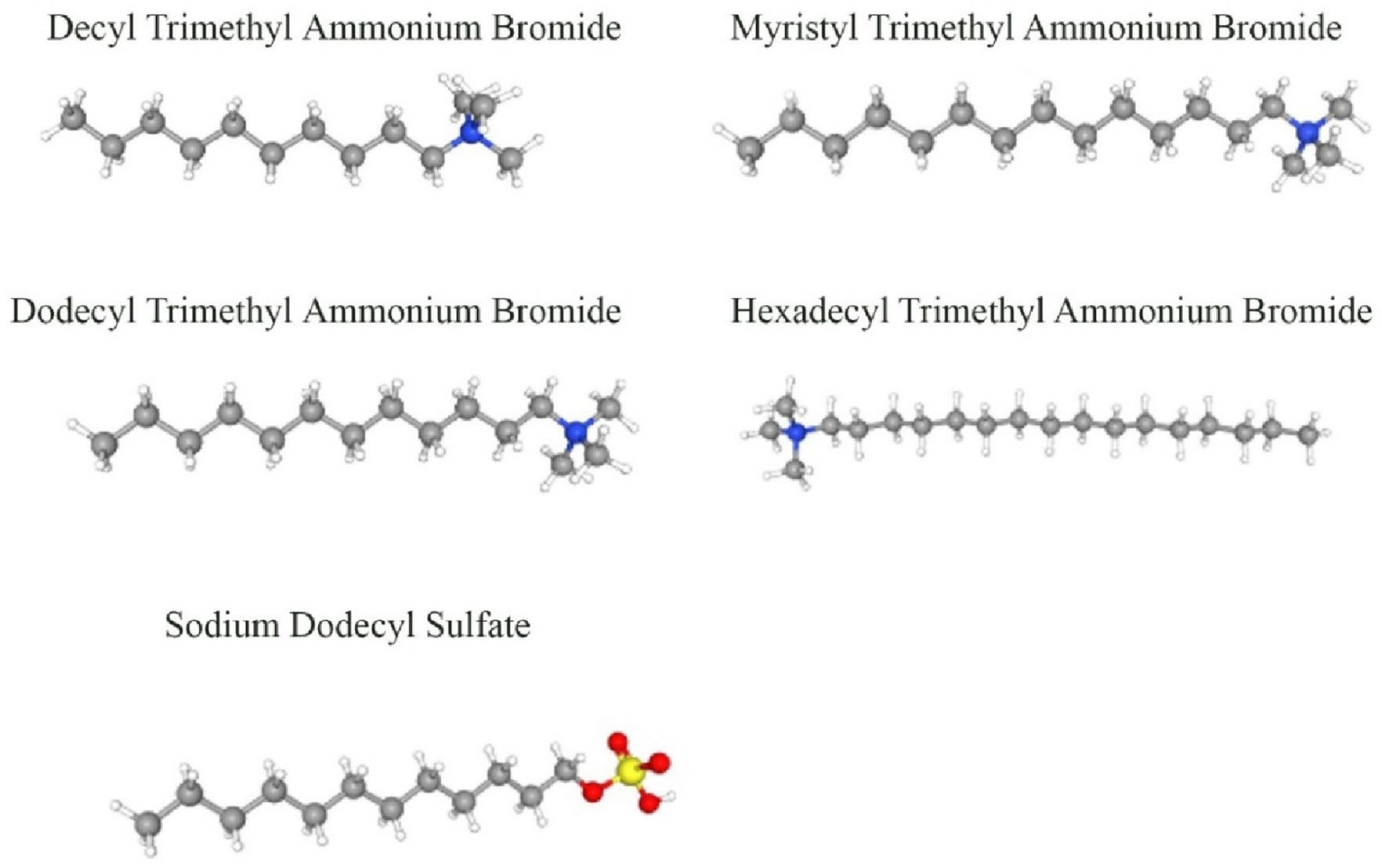
Structures of surfactants used in this study*.*

The hydrophilic group numbers and the lipophilic group numbers are obtained from tables provided by Davies (1957)^80^. The values of the HLB for the surfactants used in this study are presented in Table 1. Another parameter used to characterize the surfactant is the PIT. This parameter depends on the structure of the surfactant^76^. For better visualization, the structures of surfactants utilized in this work are depicted in Fig. 1. The amount of the PIT_x_ of the five surfactants used in this study are presented in Table 1. Moreover, the statistical parameters of the databank used in this work are represented in Table 2. The collected data were divided into training and testing categories for model development. In all modeling techniques, 80% of the sample dataset was used to train algorithms, and the remaining 20% was used to evaluate the models’ performance. At this stage, the data were randomly divided.

**Table 2:** Statistical parameters of the databank.

	HLB	dPIT/dx (°C)	Conc × 10^5^ (mol/l)	T (K)	Mw (g/mol)	IFT (mN/m)
Mean	20.86	347.87	303.35	300.01	125.05	36.64
SD	14.45	200.32	714.44	7.68	40.80	14.39
Min	0	0	0	283.15	86.178	4.98
Skewness	−0.04	−1.02	4.89	2.07	1.03	−0.68
Kurtosis	−1.09	−0.71	28.44	5.81	0.45	−0.87
Max	40	499	6896.76	333.15	240.47	53.54
Status	Input	Input	Input	Input	Input	Output

## Model development

### Decision tree

**Figure 2:**
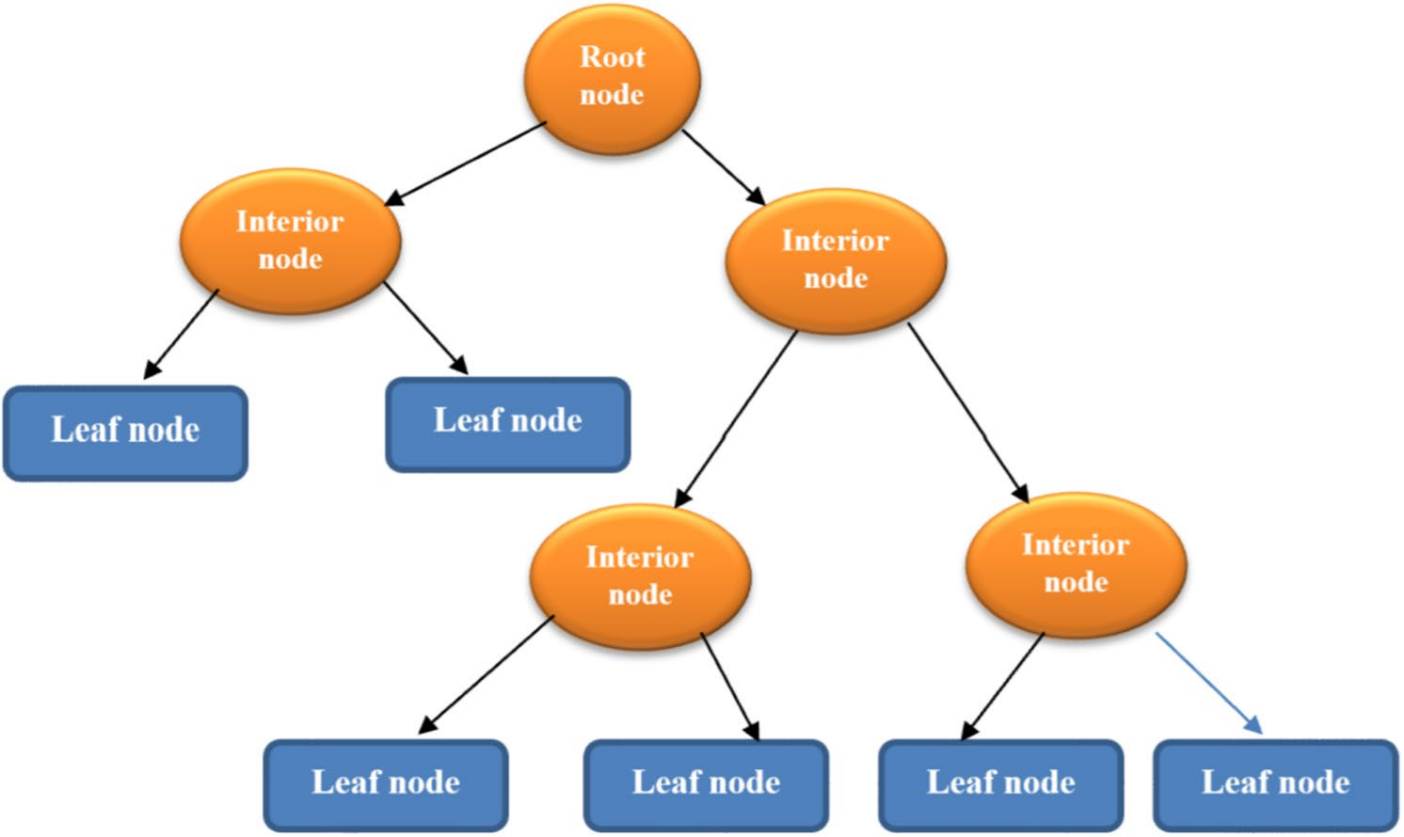
A simple structure of the DT.

The DT is used for regression and classification issues^81^. A simple structure of the DT is depicted in Fig. 2. A regression DT can predict numerical responses corresponding to independent variables. These types of algorithms are used in complex datasets. Decision trees are intuitive and interpretable^82, 83^. In decision tree regression (DTR), it constantly divides the initial input space into smaller subsets and incrementally makes the final DT with decision and leaf nodes. ID3, C4.5, C5, and Classification and Regression tree (CART) are standard DT algorithms. C4.5 is an improved version based on ID3 and has the following advantages: (1) it can work with incomplete data, (2) it can use the pruning technique to prevent over-fitting, and (3) accepting discrete and continuous features. The CART is very similar to the C4.5. The difference between the two algorithms is that the CART does not calculate the rules and can also solve regression problems^84^. In this study, the optimized version of the CART algorithm was used.

Dividing nodes in the training process of the network is one of the most critical parts of implementing a DT algorithm. In CART, it uses a Gini coefficient to divide the nodes^85^. The DT implemented in that study consists of four stages^85^. In the first stage, the DT grows using the division of nodes. After dividing the training data into two parts, with the same logic, it divides these subsets again and so on. The DT greedily searches for an optimal division. This algorithm repeats the data segmentation in each step and does not check whether it leads to less impurity in the next steps. Each node is assigned to a predicted target based on the target’s distribution in the sample data in nodes. This process continues until it cannot find a partition that reduces the impurity. Also, when the tree reaches its maximum state, the DT’s growth process will stop. It is necessary to optimize the maximum depth value for this algorithm. In the second stage, after the tree’s maximum growth, the process of building the tree stops. At this stage, the DT may not accurately predict the target value based on the test data. The third step involves pruning and simplifying the tree, which makes it better to predict. In the fourth step, the best tree is selected from the pruned trees.

The DT continuously divides the data into smaller sections during the same process to homogenize the data in the partition. The splitting rules may be set to optimize a criterion related to the target’s predictive value, or the rules may be set to minimize local node impurity or dependent variables over the training set.

Identifying the number of training data points for the DT is a significant issue because it will cause over-fit if the sample data is low. Adding any level to the tree may lead to an increase in the number of samples required to learn the DT. The size of the tree should be controlled to prevent overfitting^86^. The main parameters for DT optimization include tree depth, minimum sample division, and minimum sample leaf. Ensemble methods can prevent over-fitting in the DT algorithm^87, 88^.

### Extra trees

**Figure 3:**
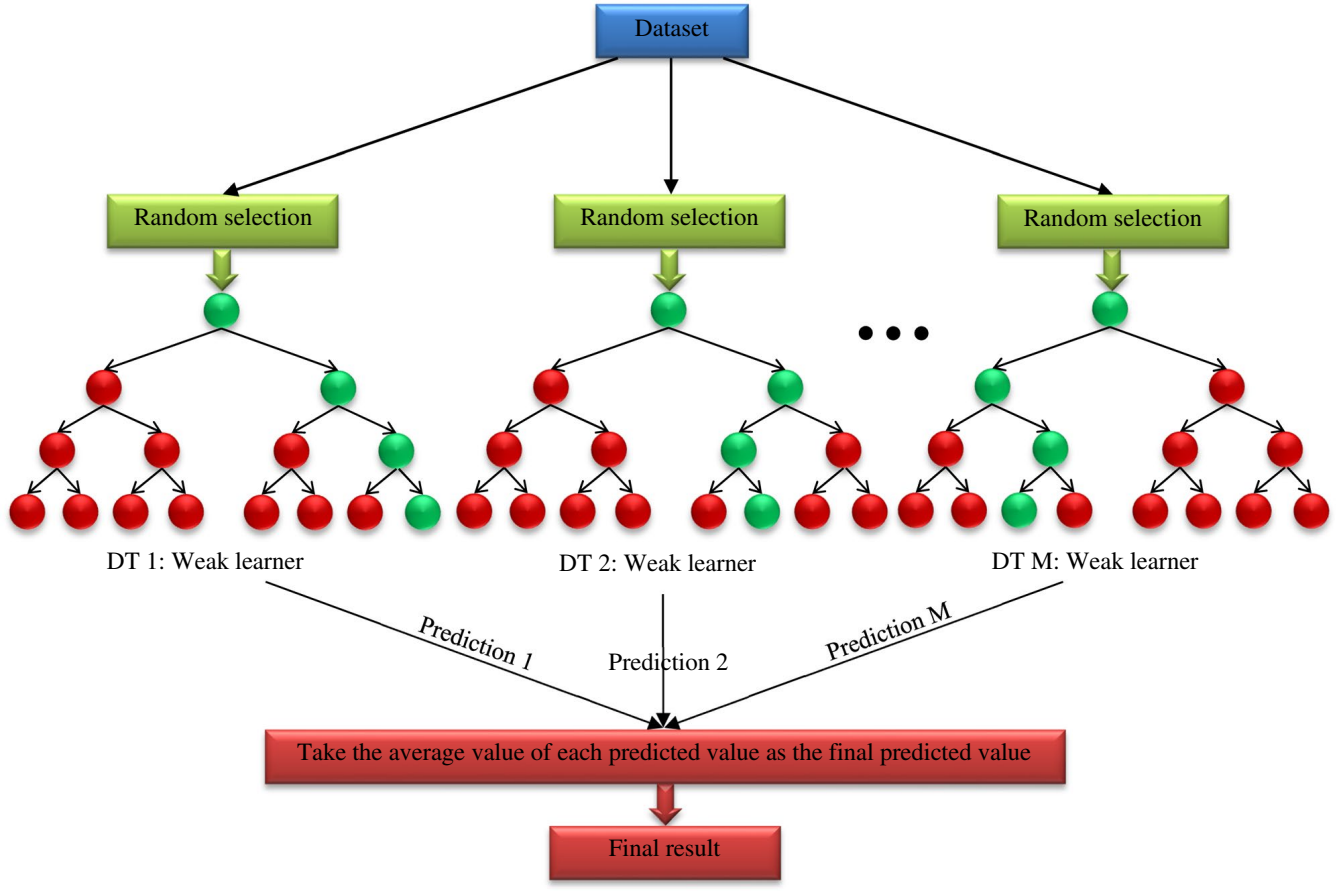
Schematic structure of the ET.

The ET creates a stronger model by combining several decision trees. The ET is one of the ensemble methods. In the ET method, the node is divided completely randomly by selecting the cutting points. Each DT grows independently, and all learning samples are used to grow the trees. The predicted target values are then added up for the final prediction. Finally, it predicts the final answer using the mathematical mean of the predicted values obtained from each base model^89^. The ET algorithm builds an ensemble model based on the explicit randomization of cutting points and feature combinations using averaging. Also, using all learning data to build base models can minimize the bias of the final model^90^. The tree growth method’s complexity in the ET algorithm, assuming the trees are balanced, is similar to other tree growth methods^89^. This algorithm has three parameters including *N*_*min*_, which denotes the minimum sample size to divide a node, *K* shows the number of randomly selected attributes in each node, and *M* illustrates the number of trees used as the base model. It is necessary to optimize these parameters to develop a more robust model based on additional trees. Each of these parameters has a different effect. The value of parameter *N*_*min*_ affects the average noise output of the model. The larger the value of *N*_*min*_, the smaller the trees are made. As a result, the variance decreases, and the bias increases. The minimum size of sample data for node splitting should be optimized according to the model’s amount of output noise. Obviously, in regression problems, high noise levels lead to overfitting. Geurts et al.^89^ suggested that a higher value for parameter *N*_*min*_ should be used to build a stronger model when the data has more noise. In other words, to optimize ET in high noise conditions, it is necessary to increase the value of *N*_*min*_. The number of selected attributes can also determine the strength of the attribute selection process. The maximum value that can be considered for *K* is the number of input features of the model. The low value of parameter *K* increases the randomness of the trees. It also makes the structure of the trees less dependent on the target value of the learning samples. Therefore, if we set the *K* to 1, the divisions are selected completely independent of the target variable. Also, if the value of this parameter is equal to the number of features in the learning data, the features are not explicitly selected randomly, and the randomization effect is applied only by selecting the cut points^89^. A schematic structure of the ET algorithm is illustrated in Fig. 3.

### Gradient boosted regression trees (GBRT)

Boosting is another method of an ensemble that combines several weak learners to create a stronger model for target prediction^85^. This method is used to solve regression and classification problems. The weak learners are trained one after the other, each focusing on correcting the previous step^91^. In this study, the GBRT was used, in which the DT is defined as the basic learner.

In a modeling issue, one has a system consisting of a set of random “explanatory” variables or “input” *x* = {× *1; …; xn*} and random “response” variables or “output” *y*. The goal is to create a function F^*^(x) that relate *y* to *x*.

3 $${\text{F}}^{*}\left({\text{x}}\right)= {\text{arg}}{}_{{\text{F}}\left({\text{x}}\right)}{}^{\text{min}}{{\text{E}}}_{\text{y, x}} {\Psi} \left(\text{y, F}\left({\text{x}}\right)\right)$$

4 $${\text{F}}\left({\text{x}}\right)= \sum_{{{\rm m}=0}}^{\text{M}}{\upbeta}_{\text{m}}{\text{h}}\left(\text{x};{\text{a}}_{\text{m}}\right)$$

the expected value of some specified loss function $\Psi \left(y,F\left(x\right)\right)$ is minimized. Here, $h\left(x;{a}_{m}\right)$ is a simple function of $x$ defined as a basic learner. The expansion coefficients $$\left\{{\upbeta }_{m}\right\}\begin{array}{l}m\\ 0\end{array}$$ and the parameters $$\left\{{a}_{m}\right\}\begin{array}{l}m\\ 0\end{array}$$ are jointly fit to the training data in a forward “stage-wise” manner.

5 $$\left({\beta}_{\text{m}}{,}{\text{a}}_{\text{m}}\right) = {\text{arg}}\,{\text{min}}_{{\beta,{\rm a}}}\sum_{{{\rm i}-1}}^{\text{N}}{\Psi}\left({\text{y}}_{\text{i}},{\text{F}}_{{\text{m}}-{1}}\left({\text{x}}_{\text{i}}\right){+\beta {\rm h}}\left({\text{x}}_{\text{i}};\text{a}\right)\right)$$

6 $${\text{F}}_{\text{m}}\left({\text{x}}\right)= {\text{F}}_{{\text{m}}-{1}}\left({\text{x}}\right){+}{\upbeta}_{\text{m}}{\text{h}}\left(\text{x};{\text{a}}_{\text{m}}\right)$$

Equation (5) can be solved in two steps for a given cost function by the Gradient Boosting method^92, 93^. First, the function $h\left(x; a\right)$ is fit by least squares:

7 $${\text{a}}_{\text{m}} = {\text{arg}}{\text{min}}_{{{\rm a},\uprho}}\sum_{{{\rm i}=1}}^{\text{N}}{\left[{\widetilde{\text{y}}}_{\text{im}}-{\uprho {\rm h} }\left({\text{x}}_{\text{i}};\text{a}\right)\right]}^{2}$$

8 $${\widetilde{\text{y}}}_{\text{im}} = -{\left[\frac{{\partial \Psi}\left({\text{y}}_{\text{i}},\text{F}\left({\text{x}}_{\text{i}}\right)\right)}{{\partial {\rm F}}\left({\text{x}}_{\text{i}}\right)}\right]}_{{\text{F}}\left({\text{x}}\right) = {\text{F}}_{{\text{m}}-{1}}\left({\text{x}}\right)}$$

In the next step, according to $$h(x;a$$_m_ $$)$$, the optimal value of $$\beta$$_Μ_ is determined. GBRT specializes in this strategy to the case where the weak learner $$h\left(x; a\right)$$ is an *L* terminal node regression tree. At each iteration *m*, a regression tree is divided the *x* space into *L*-disjoint regions $$\{R$$_lm_ $$\}$$ $$\begin{array}{l}l\\ l=1\end{array}$$.

9 $${\text{h}}\left(\text{x};\left\{{\text{R}}_{\text{lm}}\right\}\begin{array}{l}{\text{L}}\\ {1}\end{array}\right) = \sum_{\text{l-1}}^{\text{L}}{\bar{\rm y}}_{\text{lm}}{1}\left({\text{x}} \in {\text{R}}_{\text{lm}}\right)$$

10 $${\overline{\text{y}}}_{\text{im}} = {\text{mean}}_{\text{xi } \in {\text{R}}_{\text{im}}}\left({\widetilde{\text{y}}}_{\text{im}}\right)$$

the solution to Eq. (8) reduces to a simple “location” estimate based on the criterion $\Psi$.

11 $${\text{y}}_{\text{lm}} = {\text{arg}}\,{\text{min}}_{\text{y}}\sum_{{\text{x}}_{\text{i}} \in {\text{R}}_{\text{lm}}}{\Psi}\left({\text{y}}_{\text{i}}{,}{\text{F}}_{{\text{m}}-{1}}\left({\text{x}}_{\text{i}}\right)+ \text{y} \right)$$

It will be updated separately in each corresponding area.

12 $${\text{F}}_{\text{m}}\left({\text{x}}\right) = {\text{F}}_{{\text{m}}-{1}}\left({\text{x}}\right)+\upnu. {\text{y}}_{\text{lm}}{1}\left({\text{x}} \in {\text{R}}_{\text{lm}}\right)$$

**Figure 4:**
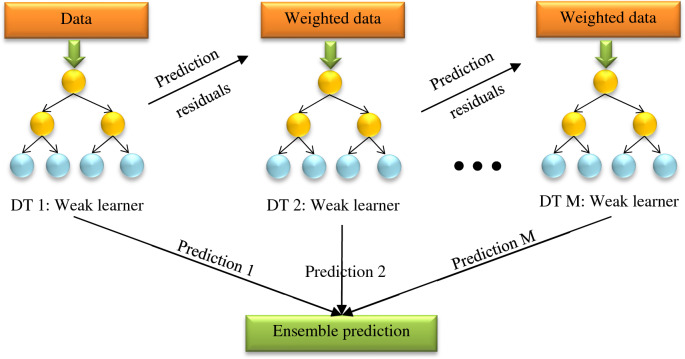
A simple architecture of the GBRT.

The learning rate is controlled by the “shrinkage” parameter $0<v\le 1$. The small values of this parameter $\left(v\le 0.1\right)$ lead to a much better generalization error. Friedman (1999)^92^ presented specific algorithms based on this template for several loss criteria, including least-absolute deviation, least squares, Huber, and for classification, *K* class multinomial negative log-likelihood. It should be noted that hyper-parameters should be considered to optimize the implemented model. These parameters such as the number of base estimators, subsample, loss function, maximum depth, the minimum number of leaf nodes, the maximum number of features, and the minimum number of sample split samples, define the structure of the network. A simple architecture of the GBRT algorithm is depicted in Fig. 4.

## Results and discussion

### Description of the models' development

In the present work, three different data-driven techniques, including DT, ET, and GBRT were developed to establish accurate models for the estimation of the IFT between ionic surfactants and normal alkanes. As mentioned, in order to create a more robust and faster model, the specific hyperparameters of each algorithm must be adjusted and optimized. As mentioned earlier, the trial-and-error method was employed to optimize the implemented models. The value of the maximum depth parameter of the DT strongly affects the speed and accuracy of the model. The depth of the tree should be carefully adjusted so as not to cause over-fitting and under-fitting. As reported in Table 3, the best value for this parameter is 7. The proposed control parameters for implementing the DT algorithm based on the sample data used in this study are reported in Table 3. In this study, two ensemble algorithms based on the DT were used. Ensemble methods were used to increase the stability and accuracy of the DT model. Ensemble models can also prevent over-fitting and create a robust and stable model based on the base estimator^94^. Due to the nature of ensemble models, the number of estimators is the most important parameter for optimization. To build an accurate model based on the GBRT algorithm, loss function, learning rate, number of estimators, subsample, maximum depth, and alpha must be considered and adjusted. The adjusted parameters for the models implemented in this study were reported in Table 3.

**Table 3:** Internal parameters of the developed models.

Model	Parameter	Value
GBRT	learning rate	0.12
	loss	Huber
	n_estimators	60
	sub_sample	0.17
	criterion	Friedman mse
	min_sample_split	2
	min_sample_leaf	1
	max_depth	9
	alpha	0.97
DT	criterion	Friedman mse
	min_sample_split	2
	min_sample_leaf	1
	max_depth	7
	ccp_alpha	0.0075
	splitter	Best
	max_features	None
ET	criterion	Friedman mse
	min_sample_split	2
	min_sample_leaf	1
	max_depth	12
	n_estimators	70
	Bootstrap	True

### Statistical evaluation

In this study, statistical criteria were used to evaluate the accuracy and ability of the developed models in predicting IFT. For this purpose, statistical parameters including determination coefficient (R^2^), average percent relative error (APRE, %), root mean square error (RMSE), standard deviation (SD), and average absolute percent relative error (AAPRE, %), were used. The formulas of these statistical parameters are listed as follows^95^:

13 $${R}^{2}=1-\frac{\sum_{i=1}^{n}{\left({IFT}_{{exp}_{i}}-{IFT}_{{pred}_{i}}\right)}^{2}}{\sum_{i=1}^{n}{\left({IFT}_{{exp}_{i}}-\overline{IFT }\right)}^{2}}$$

14 $$APRE=\frac{1}{n}\sum_{i=1}^{n}\left(\frac{{IFT}_{{exp}_{i}}-{IFT}_{{pred}_{i}}}{{IFT}_{{exp}_{i}}}\right)\times 100$$

15 $$RMSE=\sqrt{\frac{1}{n}\sum_{1}^{n}{\left({IFT}_{{exp}_{i}}-{IFT}_{{pred}_{i}}\right)}^{2}}$$

16 $$AAPRE=\frac{1}{n}\sum_{i=1}^{n}\left\lceil\frac{{IFT}_{{exp}_{i}}-{IFT}_{{pred}_{i}}}{{IFT}_{{exp}_{i}}}\right\rceil\times 100$$

17 $$SD=\sqrt{ \frac{1}{n} \sum_{i=1}^{n}{\left(\frac{{IFT}_{{exp}_{i}}-{IFT}_{{pred}_{i}}}{{IFT}_{{exp}_{i}}}\right)}^{2}}$$

If the value of R^2^ is high and the values of AAPRE, APRE, RMSE, and SD are low, the model predicts the IFT with higher accuracy. The maximum value of R^2^ is one, and the lowest value of the AAPRE value is zero. Statistical parameters for evaluating the models implemented in this study at different development stages are presented in Table 4. According to the RMSE values presented in Table 4, the accuracy of the models implemented in this study is as follows:

$${\text{GBRT}}\, > \,{\text{ET}}\, > \,{\text{DT}};{\text{ for both training and testing phases}}.$$

**Table 4:** Statistical assessment of the developed models.

		GBRT model	ET model	DT model
Train	SD	0.041	0.065	0.058
	RMSE	0.96	1.223	1.416
	APRE	−0.29	−1.32	−0.33
	AAPRE	2.47	3.17	3.53
	R^2^	0.9957	0.9945	0.9906
Test	SD	0.053	0.074	0.100
	RMSE	1.628	1.769	2.268
	APRE	−1.14	−1.28	−1.43
	AAPRE	3.63	4.89	6.46
	R^2^	0.9852	0.9827	0.9713
Total	SD	0.044	0.067	0.069
	RMSE	1.126	1.278	1.623
	APRE	−0.46	−1.32	−0.55
	AAPRE	2.71	3.52	4.12
	R^2^	0.9939	0.9925	0.9873

### Graphical error analysis

**Figure 5:**
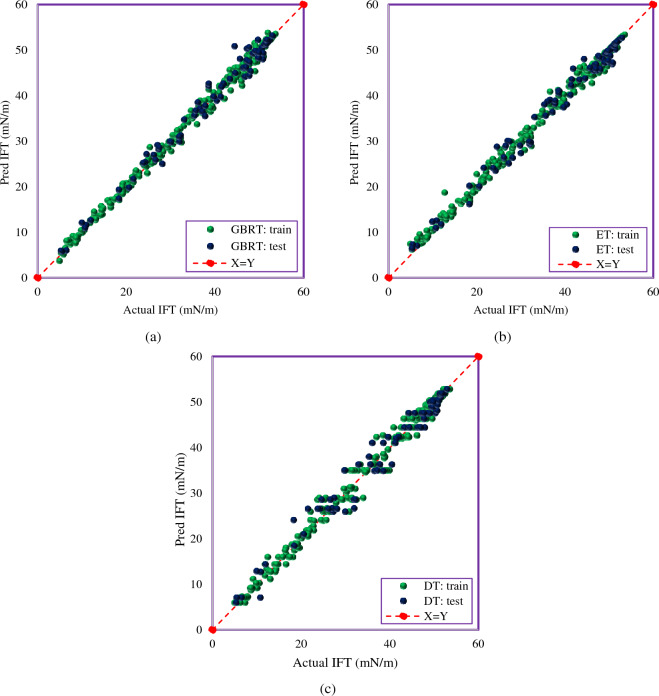
Cross plots of the developed models; (**a**) GBRT, (**b**) ET, (**c**) DT.

In this section, graphical error analysis shows the models’ validity and accuracy. Therefore, four graphs, including bar-plot, cross-plot, relative error distribution, and cumulative frequency diagram were investigated. Figure 5 is a cross-plot of DT, ET, and GBRT models. This diagram plots the predicted values in the training and testing phases versus the experimental values. In this type of diagram, if the train and test points are close to the unit slope line (X = Y), it indicates that the model can predict with high accuracy. As Fig. 5 shows, some of the test points of the DT and ET models are above or below the X = Y line, which indicates the lower accuracy of these models. Figure 5 shows that the points of the GBRT model are scattered around the unit slope line. This model estimates the IFT close to the experimental values. It can be seen that the GBRT model estimates the IFT with higher accuracy than the DT and ET models.

Furthermore, the relative error diagram is a practical tool to show the deviation of the value predicted by the model from the experimental value. Absolute error is the difference between the predicted and experimental values. The relative error is equal to the absolute error divided by the experimental value. The relative error is calculated as follows:

18 $$\text{Relative Error} \, = \text{ } \frac{{\text{IFT}}_{\text{exp}}-{\text{IFT}}_{\text{pred}}}{{\text{IFT}}_{\text{exp}}}$$

**Figure 6:**
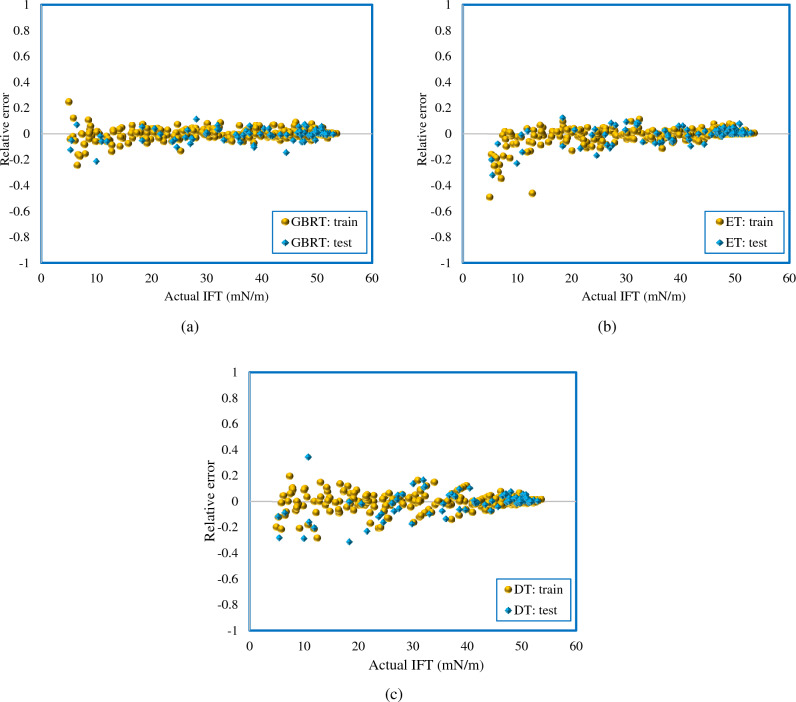
The relative deviation of the experimental results and predicted data using (**a**) GBRT, (**b**) ET, (**c**) DT models.

**Figure 7:**
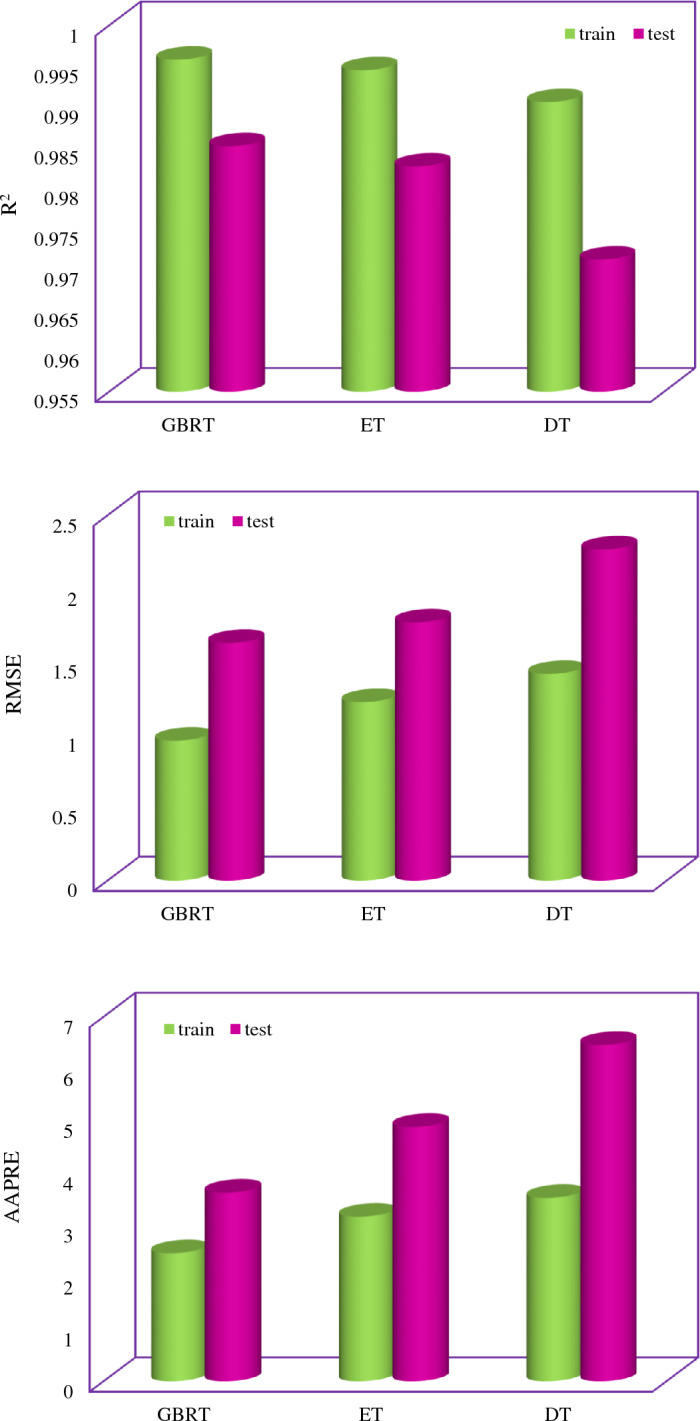
Statistical evaluation of the developed models.

In Fig. 6, the zero line indicates that the model predicts without error. Therefore, if the training and test points are close to the zero line, it indicates that a robust model has been developed. Figure 6 shows that from a value of 20 mN/m onwards, the relative error of all models implemented in this study is low. The points in the negative range of Fig. 6 with respect to the relative error Eq. (18) show that the model is overestimated. As explained in the model section, ensemble methods increase accuracy and improve overfitting^87, 96^. According to the results presented in Figs. 6 and 7, the models implemented in this study, including ET and GBRT, have reduced variance and controlled overfitting, as seen in the test phase.

**Figure 8:**
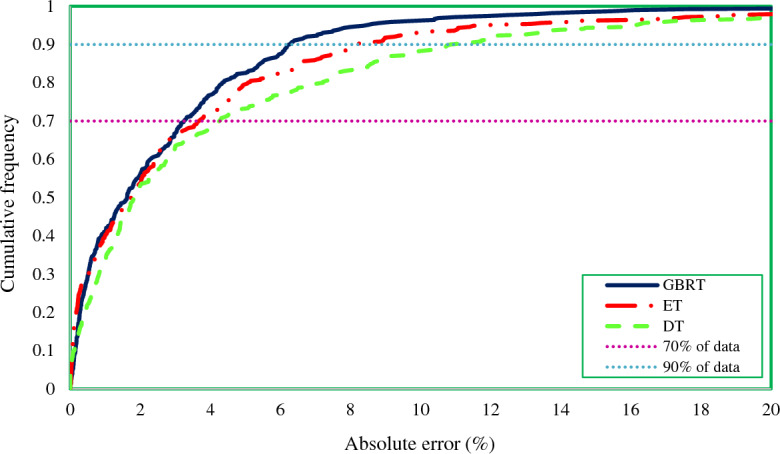
Cumulative frequency diagram of three proposed models for estimating the IFT of ionic surfactants and normal alkanes.

Next, Fig. 8 illustrates the cumulative frequency plot, which displays the proportion of predicted data that are less than or equal to a particular error value. This graph displays absolute relative errors (%) that are calculated using the next equation for different proportions of predicted data by models.

19 $$\text{Absolute }\text{Relative Error}\text{ (\%) }= \text{ } \left|\frac{{\text{IFT}}_{\text{exp}}-{\text{IFT}}_{\text{pred}}}{{\text{IFT}}_{\text{exp}}}\right|\times 100$$

The closer a model gets to the vertical axis, the more data it can predict with lower error and consequently considered a more precise model. As Fig. 8 shows, the GBRT model estimates 70% of the IFT values with just less than 3.2% error. This is while the ET and DT models have predicted this proportion of the data with an error of 3.6% and 4.2%, respectively. In addition, about 90% of the data, estimated by the GBRT model, has an error lower than 6.2%, while it is 8.2% and 10.8% for ET and DT models, respectively. As a result, the superiority of the GBRT model over the ET and DT models can be recognized again.

Based on the presented results in this section, the GBRT model is proposed to estimate the IFT between the surfactant solution and the normal alkane with high accuracy. A part of IFT values predicted by GBRT model in the test phase is reported in Table 5, and no significant difference is observed in the prediction of experimental data by this model. The results of Fig. 7 show that the AAPRE and RMSE of the GBRT model in the test phase were 3.63% and 1.628, respectively, which indicates the high reliability of this model in predicting the IFT of surfactant–hydrocarbon systems.

**Table 5:** The IFT data predicted by GBRT models in the test phase.

Number	HLB	dPIT/dx (°C)	Surfactant concentration × 10^5^ (mol/l)	T (K)	Mw (g/mol)	IFT (mN/m)	GBRT	Relative error (%)
1	0	0	0	305.65	149.29	51.26	51.05222	0.41
2	40	499	286.472	298.15	128.2	24.2583	25.39824	−4.70
3	0	0	0	310.65	170.33	51.43	51.51684	−0.17
4	40	499	837.628	298.15	240.471	10.9684	11.62199	−5.96
5	0	0	0	305.65	86.18	49.7	49.50517	0.39
6	21	338	0.100012	298.15	100.21	50.2605	49.15622	2.20
7	19	486	69.96725	298.15	114.23	24.5995	27.15528	−10.39
8	19	486	713.515	298.15	198.39	18.3707	17.41083	5.23
9	21	338	306.963	298.15	170.33	36.6211	37.08537	−1.27
10	19	486	0.010001	298.15	128.2	49.6681	52.18449	−5.07
11	19	486	9.878343	298.15	100.21	40.4008	38.8913	3.74
12	18	453	0.013639	295.15	86.18	47.2076	50.27795	−6.50
13	40	499	34.9646	293.2	86.18	39.6676	39.73671	−0.17
14	19	486	0.078221	298.15	100.21	50.7415	47.95615	5.49
15	19	486	1.91964	298.15	142.29	41.5223	42.11541	−1.43
16	19	486	98.41191	298.15	128.2	27.249	28.22018	−3.56
17	21	338	10.50997	298.15	170.33	46.6652	44.09608	5.51
18	21	338	3.009322	298.15	128.2	44.3392	50.7895	−14.55
19	40	499	8	293.2	86.178	46.4	46.08001	0.69
20	21	338	6729.21	298.15	142.29	10.0208	12.15356	−21.28
21	19	486	74.11994	298.15	170.33	31.9658	29.92372	6.39
22	40	499	14	303.2	86.178	43.3	43.61458	−0.73
23	21	338	31.75242	298.15	170.33	41.8647	42.63048	−1.83
24	0	0	0	313.15	100.21	49.38	49.48231	−0.21
25	19	486	0.971186	298.15	114.23	47.7117	44.35099	7.04
26	21	338	102.32	298.15	86.18	36.0927	38.4876	−6.64
27	40	499	0	288.2	86.178	51.4	51.62253	−0.43
28	40	499	672.085	298.15	142.29	11.9565	12.68166	−6.07
29	0	0	0	310.65	114.23	50.09	49.88087	0.42
30	40	499	793.492	298.15	86.18	6.51163	6.071484	6.76
31	18	453	42.27935	295.15	86.18	18.3688	19.35079	−5.35
32	0	0	0	328.15	128.2	49.09	49.23298	−0.29
33	40	499	679.088	298.15	114.23	10.7708	10.98328	−1.97
34	21	338	1030.93	298.15	170.33	27.0355	29.14769	−7.81
35	40	499	96.628	298.15	86.18	27.4419	28.60547	−4.24
36	21	338	298.274	298.15	128.2	35.342	35.62043	−0.79
37	21	338	9.56027	298.15	142.29	47.9878	45.3272	5.54
38	40	499	169.492	298.15	114.23	29.8419	29.91685	−0.25
39	0	0	0	298.15	156.31	52.25	52.14065	0.21
40	40	499	83.55376	298.15	114.23	37.1542	36.40291	2.02

### Sensitivity analysis

**Figure 9:**
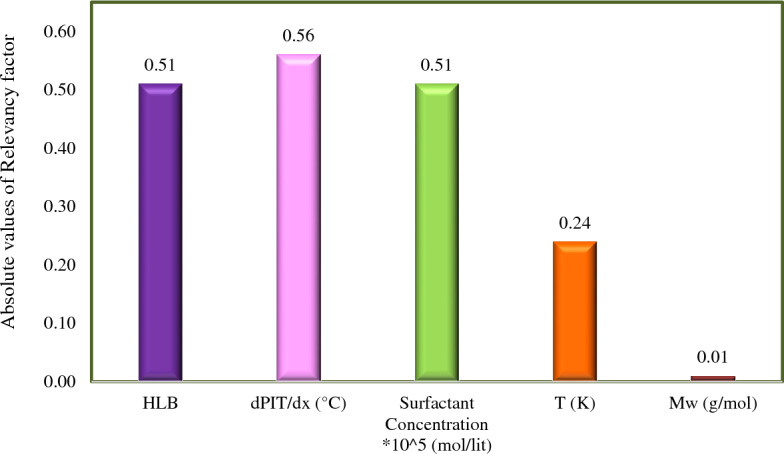
The absolute effect of each input parameter on the IFT of surfactant–hydrocarbon systems based on Pearson correlation coefficient.

In this study, the sensitivity analysis was performed to determine the extent and type of relationship between the independent variables presented in Table 2 and the amount of the IFT (output). Different methods of sensitivity analysis are introduced for regression models^97, 98^. In this section, the Pearson coefficient was used to calculate the relevancy factor^99^:

20 $$\text{RF} = \frac{\sum_{\text{i=1}}^{\text{n}}\left(\overline{{\text{x} }_{\text{k}}}-{\text{x}}_{\text{k. i}}\right)\left(\stackrel{\mathrm{-}}{\text{y}}-{\text{y}}_{\text{i}}\right)}{\sqrt{\sum_{\text{i=1}}^{\text{n}}{\left(\overline{{\text{x} }_{\text{k}}}-{\text{x}}_{\text{k. i}}\right)}^{2}\sum_{\text{i=1}}^{\text{n}}{\left(\stackrel{\mathrm{-}}{\text{y}}-{\text{y}}_{\text{i}}\right)}^{2}}}$$

where *n* and *k* represent the number of data points and the type of input variable. The symbols *y*_*i*_, $\stackrel{\mathrm{-}}{\text{y}}$, *x *_*k.i*_, and *x*_*k*_ denote the target, the average of the target value, the input value, and the *k*_th_ input value average. Figure 9 shows the absolute values of sensitivity analysis results of the proposed GBRT model. In this data set, PITx, the surfactant concentration, and HLB had the greatest effect on IFT, respectively. At concentrations lower than the CMC of surfactants, the IFT decreases with increasing surfactant concentration^100^. Therefore, the surfactant concentration was expected to  have large effect on IFT along with the type of surfactants. The molecular weight of alkanes has the lowest value of the Pearson coefficient compared to other input parameters.

### Trend analysis

**Figure 10:**
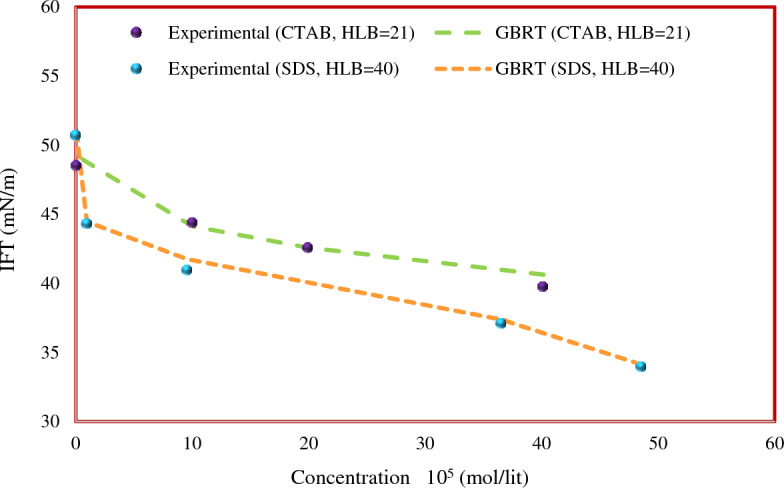
Comparison of predicted and experimental IFT trends for the systems of *n*-hexane and surfactants C_10_TAB and SDS at 298.2 K.

In this section, the ability of the proposed GBRT model to predict IFT behavior in different conditions was investigated. Figure 10 shows the predicted values of IFT by the GBRT model and the experimental data^68, 69,72^. In this figure, the IFT of anionic (SDS) and cationic (C_10_TAB) surfactants with n-hexane as hydrocarbon phase was plotted. The temperature of the surfactant solution was considered constant (298.2 K), and IFT data was plotted as a function of surfactant concentration. Based on the results presented in Fig. 10, it can be seen that the GBRT model accurately predicts the IFT of the surfactants and n-hexane systems. The IFT between the surfactant solution and the hydrocarbon depends on the surfactant concentration^101–103^. At concentrations lower than the CMC, by increasing the surfactant concentration, the IFT value will reduce^38^. Surfactant molecules are adsorbed on the liquid–liquid interface and reduce IFT^104^. Therefore, increasing the adsorbed surfactant molecules at the interfacial boundary further reduces the IFT.

**Figure 11:**
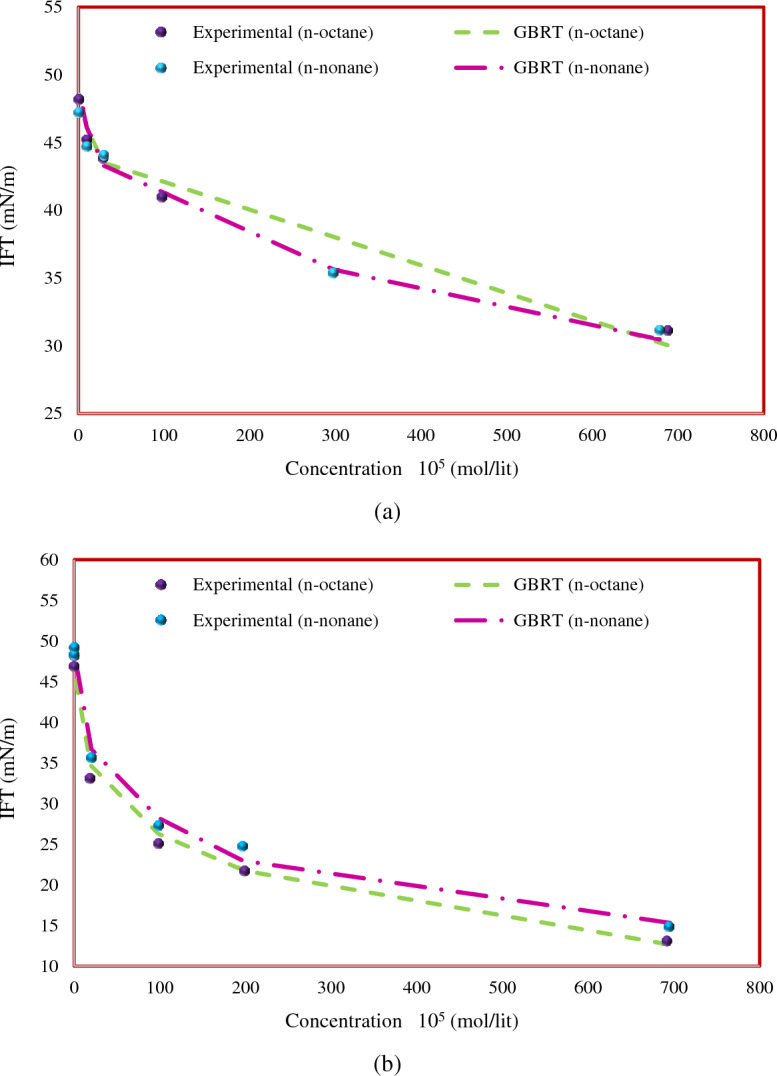
Comparison of predicted and experimental IFT trends for pure hydrocarbons at 298.2 K; (**a**) C_10_TAB and (**b**) C_12_TAB.

In the next step, Fig. 11 shows the IFT between C_10_TAB and C_12_TAB in n-octane and n-nonane hydrocarbon phases^69^ along with the prediction of the GBRT model. For this analysis, the temperature was considered a constant value of 298.2 K. As can be seen, the heavier the hydrocarbon phase, the greater the IFT of surfactant–hydrocarbon. Again, the GBRT model renders great predictions for the IFT of the surfactants and hydrocarbons considered in this figure.

**Figure 12:**
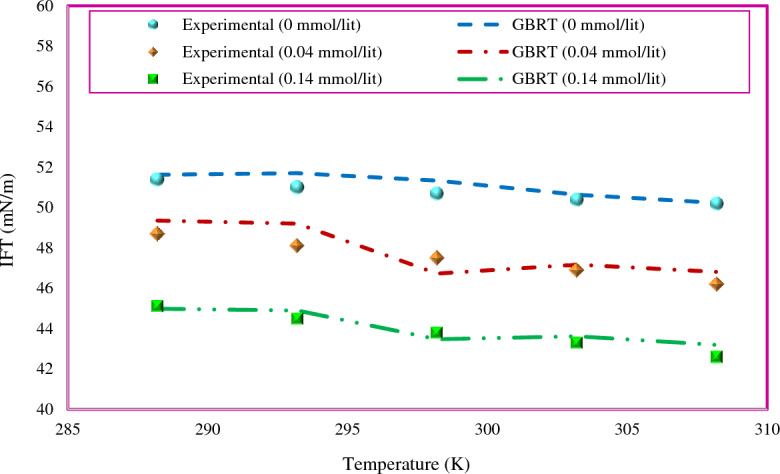
Comparison between experimental and predicted IFT values by the GBRT model for SDS surfactant in n-hexane mixture at different temperatures and concentrations.

Another parameter affecting the IFT of the surfactant that was considered in this study is temperature. The IFT values were predicted for different concentrations of SDS corresponding to a temperature range. Using the GBRT model, a comparison of the predicted trend of IFT changes in the temperature range with experimental data^70^ is shown in Fig. 12. The predicted trend is similar to the experimental data and also shows good agreement with the real trend of IFT variation. As mentioned earlier, the IFT decreases with increasing temperature. It also shows that the proposed GBRT model predicts the interfacial behavior of the surfactant well under defined conditions.

### Outlier detection using Leverage approach

The Leverage approach^105–107^ is a reliable technique to discover outliers that may exist in a databank due to a variety of circumstances, including experimental errors. These points are located at an improper distance from the majority of data. Therefore, catching the inappropriate data noted above is critical for preventing model inaccuracy and unreliability. According to the Leverage method, the values of the standardized residuals (*R*) as well as a matrix named the Hat matrix, which is made up of the exploratory and predicted values obtained from the model, are needed to conduct this analysis. The leverage or Hat indexes (*H*) are determined using the following formula^108–110^:

21 $$H=X({{X}^{T}X)}^{-1}{X}^{T}$$

Here, *X* represents the matrix of explanatory variables, and *T* represents the transpose matrix operator. Moreover, the critical Leverage (H*) is calculated according to the following formula^111^:

22 $${H}^{*}=\frac{3(number\,of\,inputs+1)}{number\,of\,data\,points}$$

In this work, the databank includes four inputs and 390 data points, leading to H* = 0.0461. On the other hand, considering *MSE* as the mean square of error, *e*_*j*_ as the error value of the *j*th data, and H*j* as the *j*th Leverage value, *R* values can be determined as follows^90, 112^:

23 $${R}_{j}=\frac{{e}_{j}}{{[MSE\left(1-{H}_{j}\right)]}^{0.5}}$$

**Figure 13:**
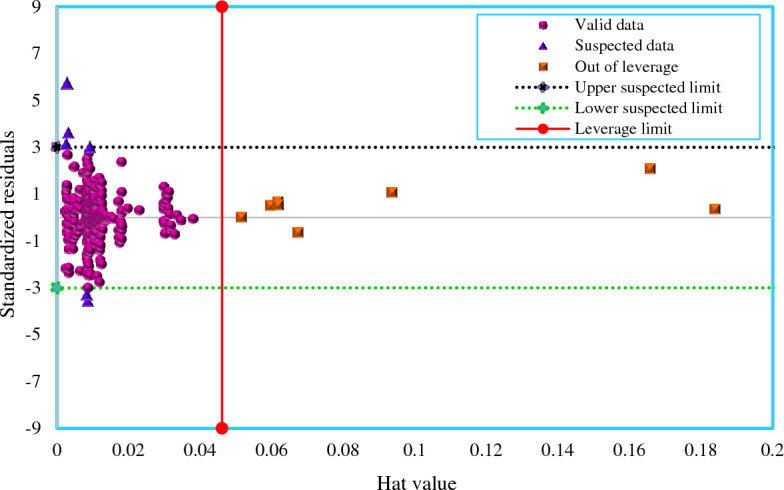
William's plot of the developed GBRT model.

If the most of the data points are positioned in the ranges of *−*3 ≤ *R* ≤ 3 and *0* ≤ *H*_*j*_ ≤ H*, both the experimental data and the model's estimations will be statistically trustworthy and precise^113^. William's plot shows *R* values versus *H* values to identify outliers. Figure 13 displays the outcomes of the leverage approach utilizing the GBRT model's results. In this case, only 6 points are recognized as suspected data, which are located outside of the model application scope. Also, it was found just 8 points as outliers. This confirms that the experimental database of IFT between surfactants and hydrocarbon is highly reliable, and the GBRT model is statistically dependable and valid.

## Conclusions

The aim of this study was to develop accurate and reliable models to estimate the IFT of ionic surfactants and normal alkanes. In this study, three intelligent computer-aided algorithms namely DT, ET, and GBRT models were implemented for this purpose. A databank containing 390 experimental IFT data points presented in the literature was used to develop these models. The models considered the following parameters as input: temperature, normal alkane molecular weight, surfactant concentration, HLB, and PIT. Based on this work, the following conclusions are drawn:

1. The ensemble methods implemented in this study, GBRT and ET, were able to reduce the variance of the DT model.
2. Among all the models developed in this study, the GBRT was the best model for predicting the IFT between the surfactant and normal alkanes.
3. Statistical evaluation in the test phase showed that the AAPRE% and RMSE of the GBRT model are 3.63% and 1.628, respectively.
4. The trend analysis demonstrated that the predictions of the GBRT model follow the expected variations in terms of the independent variables.
5. The cumulative error distribution of the GBRT model was very satisfactory, with approximately 90% of the predicted data having a relative error of less than 6.2%.
6. According to the results of the sensitivity analysis, the effect of input parameters on the IFT is as follows: PIT > surfactant concentration > HLB > temperature >  molecular weight of normal alkane.
7. The Leverage method demonstrated that the majority of the data points (almost 96.5%) are valid and both the IFT databank and the GBRT model seem to be highly trustworthy.

As a suggestion for future studies, it can be mentioned that the simultaneous involvement of the aqueous phase containing surfactant, the organic hydrocarbon phase and the solid phase including reservoir rock or its minerals can correlate the governing equations in the area of surface wetting.

## Data Availability

The datasets used during the current study are available from the corresponding author on reasonable request.
